# The relationship between professional self-concept and work-related quality of life of nurses working in the wards of patients with COVID-19

**DOI:** 10.1186/s12912-021-00595-2

**Published:** 2021-05-12

**Authors:** Akram Farhadi, Razieh Bagherzadeh, Aynaz Moradi, Reza Nemati, Leila Sadeghmoghadam

**Affiliations:** 1 Department of Health Education and Health Promotion, Bushehr University of Medical Sciences, Bushehr, Iran; 2 Department of Midwifery, Faculty of Nursing and Midwifery, Bushehr University of Medical Sciences, Bushehr, Iran; 3 Bone and Joint Diseases Research Center, Chamran Hospital, Shiraz University of Medical Sciences, Shiraz, Iran; 4 Department of Medical Emergencies, School of Allied Medical Sciences, Bushehr University of Medical Sciences, Bushehr, Iran; 5 Department of Nursing, School of Nursing, Social Development and Health Promotion Research Center, Gonabad University of Medical Science, Gonabad, Iran

**Keywords:** COVID-19, Job satisfaction, Job stress, Nurse, Professional self-concept, Work-related quality of life

## Abstract

**Background:**

Nurses are at the forefront of providing health care services and their performance is largely determinant of the quality of health care. This study aims to investigate associations between professional self-concept (PSC) and WRQoL among nurses from selected hospitals in Bushehr and Shiraz cities (south of Iran), during the period of COVD-19 pandemic.

**Method:**

This study is designed as a cross-sectional study. Available sampling was performed among active nurses in the care wards of patients with Covid-19 in public hospitals in Bushehr and Shiraz. Data were collected using demographic information form, along with the work-related quality of life and professional self-concept questionnaires. SPSS software and univariate and multivariate linear regression statistical methods with a significance level of 0.05 were used to analyze the data.

**Results:**

The mean scores of the PSC and the WRQoL Scale in nurses were respectively 202.32 ± 38.19 and 68.81 ± 19.12. There was also a significant direct relationship between PSC and WRQoL. PSC together with work location and working experience could thus explain 34.6% of the variance in WRQoL, which was 26.5% for PSC.

**Conclusion:**

Considering the confirmation of the predictive role of nurses’ PSC in their WRQoL in terms of planning and designing interventions to boost their WRQoL, attention to internal factors such as PSC is of utmost importance.

## Introduction

As integral parts of health systems, nurses are the largest community of professionals at the frontline of service delivery [1]. As reported in statistics, half of the health care services are provided by nurses [2]. The capabilities of nurses in other roles related to health care services such as counseling, management, education, research, communication, and support have further doubled the significance of their positions as health care providers [3]. Despite the vital role of nurses in providing health care services to patients and improving their quality of life, their own personal needs and quality of life have been thus far neglected [4, 5].

Work-related quality of life (WRQoL) is a complex and broad concept that has not been precisely defined so far [6]. It can be a new form of job satisfaction to the extent that the members of an organization can meet their personal needs through their own experiences within an organization. WRQoL also encompasses individuals’ feelings about all aspects of their working life including financial rewards and monetary incentives, occupational benefits, job security, comparison of working conditions with those of employees involved in other organizations, promotion opportunities, decision-making power, as well as relationships with co-workers and organizations [7]. Employees’ satisfaction and WRQoL can also directly influence the capacities of organizations in terms of proper service delivery to customers [8]. The results of studies in this line have revealed that more than half of nurses have reported their WRQOL at moderate-to-lower levels [4, 5, 9]. According to these surveys, nurses’ WRQoL has been influenced by internal and external factors such as sociocultural conditions, types of jobs, individuals’ perceptions of job satisfaction, working conditions, salary and benefits, workplace management system (WMS), as well as some demographic variables Including gender [4, 5, 9, 10].

One of the factors related to job satisfaction and WRQoL of nurses is their perceptions of job identity [10, 11]. In this respect, professional self-concept (PSC) is a multidimensional concept, which indeed refers to individuals’ cognition and understanding of themselves as professionals, affecting their way of thinking, role development, as well as professional behavior and performance [11, 12]. Among different professions, some need higher degrees of self-concept, and nursing has a special position in this regard [11, 13]. According to Kim and Choi, positive PSC and critical thinking can boost nurses’ abilities in solving problems and improving nursing care services [14]. Within nurses’ stressful work environments, PSC can also play an important role in terms of better adaptation to working conditions, reduction in job stress, and higher levels of job satisfaction [11, 13, 15]. With reference to related studies, the concept of PSC can additionally affect job outcomes such as WRQoL and burnout. Besides, it can be modified through individual and environmental interventions, since it is formed during daily experiences in the workplace [11, 15].

Following the current outbreak of coronavirus disease (COVID-19) all over the world, including Iran, the World Health Organization (WHO) has declared it as a public health emergency of international concern [16]. Nurses, working at the frontline of primary care and treatment, are thus involved in conditions different from normal ones prior to this pandemic. Isolation, working under high-risk conditions, contacts with infected individuals, long working hours, being away from home, and stress, all have led to changes in their working conditions as well as their health status and attitudes [17]. As claimed by the results of recent studies, working at the frontline during COVID-19, compared with other behind-the-scenes jobs can be associated with increased mental health problems [18, 19]. According to some studies conducted in critical situations such as the Covid-19 pandemic, nurses’ professional self-concept has improved, and this, in turn, can have consequences such as better quality of work life and nurses’ stay in their profession despite high-risk conditions [20, 21] .

Given that Iran is among the top 10 countries in the prevalence of Covid virus 19 [22], and concerning that a very high percentage of nurses in the health care system are women, who in addition to their official job are required to perform other tasks related to their role as women in traditional society, like many other Asian countries, and in this respect, there are significant differences with developed countries, the study of these variables in these specific socio-cultural conditions seems necessary [23]. Besides, in these critical situations for reasons such as the high importance of nurses’ role in the provision of services in health care systems, and the variability of factors such as professional self-concept in these special situations and its impact on nurses’ perception of the quality of work life and its dimensions, the study of the status of these nurses in terms of WRQoL and related factors is of great importance [20, 21]. Awareness of the impact of different dimensions of professional self-concept on WRQoL will pave the way for designing interventions related to those dimensions, and by doing so, to keep nurses in health care systems longer and with better quality in these critical conditions and other similar cases. Examining the status of nurses working under these critical conditions, in terms of WRQoL and related factors affecting hospital care and patients’ quality of life, is of utmost importance. The research gap in the Iranian nursing community also duplicates this necessity. This study aimed to investigate the relationship between PSC and WRQoL among nurses working in selected hospitals in Shiraz and Bushehr, in the south of Iran, during the current outbreak of COVID-19.

## Methods

### Design and participants

This cross-sectional study was performed in selected hospitals located in Shiraz and Bushehr, two southern cities in Iran, considered as the referral centers for patients with COVID-19. The study population consisted of nurses working in these centers, recruited using convenience sampling method from early May to late June 2020.

The inclusion criteria were signing informed consent forms to participate in the study, having no psychiatric disorders, and taking no psychotropic medications (as self-reported by the participants). In order to determine the sample size, and given that no studies encompassing both concepts (i.e. PSC and WRQoL) were found, the correlation coefficient of clinical competency, which was similar to the given concept, in the survey by Mokhtari et al. [24], was used. In view of the correlation coefficient of 3.0, Cronbach’s alpha of 0.05, and test power of 0.80, the sample size was considered 138 individuals, employing the G*Power software (version 3.1.9.2). Since regression analysis was finally used to analyze the data, the sample size was considered for regression according to the sample size formula, i.e., 10 to 30 variables per predictor variable. Due to having about eleven predictor variables (namely, one main variable and 10 demographic ones), the sample size was determined by 220 individuals, which was finally 264 cases after taking account of 20% attrition. Since the sample size was close to the total number of the nurses working in the hospital wards for COVID-19 patients, the questionnaire was given to all the nurses meeting the inclusion criteria and ultimately 263 individuals completed and returned it. The data collection was also conducted online. In this respect, the nurses working in the hospital wards for patients with COVID-19 were invited to participate in the study by the head nurse through the convenience sampling method.

### Data collection

To collect the data, the demographic characteristics information form (including age, gender, level of education, marital status, working experience, employment status, working hours per week, and work location) together with the Nurse Self-Concept Questionnaire (NSCQ) and the Work-Related Quality of Life (WRQoL) Scale were administered. The questionnaires were designed through web-based software, sent to the nurses via smartphone, and were then completed in a self-administered manner.

#### Nurse self-concept questionnaire (NSCQ)

The NSCQ [12] included 36 items, whose responses were based on an eight-point Likert-type scale (totally false, false, relatively false, somewhat false, somewhat true, relatively true, true, totally true) and scored from 1 to 8, with the total score ranged from 36 to 288. Accordingly, a higher score indicated a higher level of PSC in nurses [12]. Badieh Peyma Jahromy et al. (2014) have also confirmed the construct validity of this questionnaire through exploratory factor analysis in Iran. Moreover, they have measured the reliability of the questionnaire concerned using the split-half method and internal consistency, wherein the Spearman-Brown prophecy formula and Cronbach’s alpha coefficient were reported respectively 0.84 and 0.97 [25].

#### Work-related quality of life (WRQoL)

The WRQoL Scale [26] was comprised of 32 items and seven subscales, i.e., job and career satisfaction (items no. 1, 3, 8, 11, 18, and 20), working conditions (items no. 13, 16, 22, and 31), general well-being (items no. 4, 9, 10, 15, 17, and 21), home-work interface (items no. 5, 6, 14, and 25), stress at work (items no. 7, 19, 24, and 29), control at work (items no. 2, 12, 23, and 30), and work involvement (items no. 26, 27, and 28), whose responses were based on a four-point Likert-type scale (4 = totally disagree to 1 = totally agree). Item 32 in the questionnaire examines the overall Work-Related Quality of Life. In Iran, Mazloumi et al. (2017) have already confirmed the validity and reliability of this questionnaire for nurses. According to the results for reliability, the total score using Cronbach’s alpha coefficient had been reported 0.921 [7].

### Data management and analysis

The data were also analyzed using the SPSS Statistics software (version 19) along with descriptive statistics (viz. frequency, mean, and standard deviation: SD) and inferential statistics (univariate and multivariate linear regression). First, univariate regression was performed to determine the relationship between the main variable of PSC, as well as demographic variables and WRQoL. Then, to determine the relationship between the PSC variable and the WRQoL with adjustment in terms of other variables, multivariate linear regression was created. Variables that were related to the work-related quality of life in univariate regression were entered into multivariate regression. It should be noted that multiple regression variables, creating a multiple linear regression, were not imported simultaneously. For example, with regard to age and working experience, the former was entered into the regression. Also, the assumptions of the linear regression were the normal distribution of residuals, independent residuals, no observation in which dependent or independent data were assumed as outliners, and establishment of no multicollinearity. For all cases, a significance level lower than 0.05 was also considered.

### Ethical consideration

This study was a proposal approved by the Research Council of Bushehr University of Medical Sciences with the ethical identification number of IR.BPUMS.REC.1398.015. In this study, ethical considerations such as obtaining an informed consent form from every participant, and the principles of confidentiality and anonymity were observed.

## Results

The data obtained from 263 nurses working in selected hospitals based in the cities of Bushehr and Shiraz, southern Iran, were finally analyzed. Accordingly, the mean ± SD of the participants’ age was 33.32 ± 7.46 years (range: 32–58 years) and the average working experience was 9.38 ± 7.18 years (range: 1–29 years). As well, the mean ± SD of working hours per week was 51.73 ± 22.80 h (range: 6–84 h). The majority of the participants were female (81.7%). Most of the participants had a Bachelor’s degree (83.7%). In terms of marital status, 63.5% of the participants were married. Moreover, the participants with a permanent employment status accounted for 47.5% of the cases (Table 1).

**Table 1:** Demographic characteristics of nurses (*n* = 263)

Variable	Categories	Mean ± SD	
Age		33.32 ± 7.46	
Working experience		9.38 ± 7.18	
Working hours per week		51.73 ± 22.80	
		**Percentage**	**No.**
Work location	Shiraz	80.6	212
	Bushehr	19.4	51
Gender	Male	18.3	48
	Female	81.7	215
Level of education	Diploma	6.1	16
	Bachelor’s degree	83.7	220
	Master’s degree	10.3	27
Marital status	No spouse	36.5	96
	Living with one’s spouse	63.5	167
Employment status	Permanent	47.5	125
	Project-based	32.7	86
	Casual	5.7	15
	Limited-term	6.8	18
	Contractual	7.2	19

The mean scores for the WRQoL Scale and the NSCQ as well as their subscales are presented in Table 2. Nearly 70% of the nurses (*n* = 187) obtained the WRQoL Scale scores less than the median. In terms of ranking the WRQoL, the dimensions ranked from the highest to the lowest score as follows: job and career satisfaction, control at work, general well-being, work involvement, working conditions, home-work interface, and stress at work were respectively assigned with higher to lower median. Job and career satisfaction with the mean ± SD of 15.06 ± 4.25 and work involvement with the mean ± SD of 6.60 ± 2.50 obtained the highest and the lowest scores, respectively (Table 2).

**Table 2:** Mean and SD of Work-Related Quality of Life, professional self-concept, and their subscales (*n* = 263)

Variable	Subscales	SD	Mean
**Work-Related Quality of Life**	Job and career satisfaction	4.25	15.06
	Working conditions	3.21	8.27
	General well-being	4.51	13.43
	Home-work interface	3.25	8.57
	Stress at work	2.09	7.65
	Control at work	3.19	9.22
	Work involvement	2.50	6.60
	QWL total score	19.12	68.81
**professional self-concept total score**		38.19	202.32

According to the univariate regression analysis, age, working experience, work location, and PSC scores were correlated with WRQoL (Table 3).

**Table 3:** Univariate regression analysis to determine variables related to Work-Related Quality of Life in nurses (*n* = 263)

Variable	Categories	Significance level	Standardized regression coefficient
**Age**		< 0.001	0.245
**Working experience**		0.001	0.214
**Working hours per week (prior to COVID-19 pandemic)**		0.246	0.072
**Work location: Shiraz (Reference: Bushehr)**		< 0.001	0.251
**Female nurses**		0.240	−0.073
**Level of education (Reference: Diploma)**	Bachelor’s degree	0.811	−0.023
	Master’s degree	0.373	0.086
**Living with one’s spouse**		0.303	0.064
**Employment status (Reference: Permanent)**	Project-based	0.090	−0.112
	Casual	0.914	0.007
	Limited-term	0.429	−0.051
	Contractual	0.499	−0.043
**professional self-concept total score**		< 0.001	0.572

The results of multivariate regression analysis additionally showed that age and work location were placed in the first model (that is, age was not entered into the regression due to multicollinearity with working experience). These two variables together could predict 2.8% of the variance in WRQoL. In the second model, in which PSC was supplemented, 26.5% was added to the explained variance that was statistically significant (F-change =105.055, *p*-value < 0.001). The variables in the second model (namely, working experience, work location, and PSC) could also explain 34.6% of the WRQoL variance. With regard to the second model, all the variables were in the model and work location (i.e., Shiraz) and PSC had a statistically direct correlation with WRQoL. However, working experience, which was related to WRQoL in the first model, had no relationship with WRQoL after entering PSC (Table 4).

**Table 4:** Multivariate regression analysis coefficients to examine predictors of QWL (*n* = 263)

	Model 1					Model 2				
	Non-standardized regression coefficient (B)	Standardized regression coefficient (Beta)	Significance level (*P*-value)	Confidence interval 0.95%		Non- standardized regression coefficient (B)	Standardized regression coefficient (Beta)	Significance level (*P*-value)	Confidence interval 0.95%	
				Lower bound	Upper bound				Lower bound	Upper bound
Y-intercept	52.474	–	.000	45.198	59.750	5.031	–	.368	−5.959	16.020
Working experience	.444	.166	.007	.123	.766	.093	.035	.511	−.186	.373
Work location: Shiraz (Reference: Bushehr)	10.232	.212	.001	4.424	16.040	6.966	.144	.006	2.026	11.907
professional self-concept	–	–	–	–	–	.270	.540	.000	.218	.322
Coefficient of determination (R^2^)	0.089					0.354				
Adjusted R^2^	0.082					.0346				
F-change	12.489					46.724				
*P*-value	< 0.001					< 0.001				

Since age was not related to QWL in the univariate analysis, it was not included in the analysis due to multiple alignments with working experience

## Discussion

This study aimed to examine the relationship between PSC and WRQoL among nurses working in hospital wards for patients with COVID-19 in selected hospitals located in the cities of Shiraz and Bushehr, in the south of Iran. To the best of the authors’ knowledge, no study has so far explored this relationship in the Iranian nursing community. Most surveys have been merely performed in Western countries with different cultures and socioeconomic structures. Concerning the role of these factors in influencing individuals’ perceptions of their profession and its consequences, the results of the given studies could not be extended to nurses working in Iran. Innovation and fulfillment of this research in such a critical time were thus among the strengths of the present study.

The results of this study correspondingly demonstrated that two-thirds of the nurses had poor WLQ. Among the WLQ subscales, job and career satisfaction and stress at work have obtained the highest mean scores and the home-work interface has assigned with the lowest mean score. Other studies among Iranian nurses working in tertiary hospitals in Tehran [5], those involved in the city of Kashan [9], and the ones operating in intensive care units (ICUs) in Tehran [4], had further revealed that more than half of the nurses had reported their WRQoL at moderate-to-low levels. Kelbiso et al. (2017), in a study on nurses working in hospitals and health care centers in Nigeria further reported that WRQoL in two-thirds of the cases had been at an unsatisfactory level [27], which was in line with the results of the present study. It should be noted that quality of life can be under the influence of numerous factors such as salary and benefits, demographic variables, occupational safety and health events, work-related stress, job security and discipline in the workplace, adjusted hygiene conditions, basic amenities, and job prospects [4, 5, 9]. Therefore, low WRQoL in these nurses can be considered a consequence of the current critical situation at the time of COVID-19 outbreak and the increase in work pressure and stress induced by unidentified aspects of the disease.

As mentioned, the subscale of job and career satisfaction received the highest mean score with items encompassing concepts related to the existence of opportunities for progress and job promotion, training courses, chances to use one’s ability, as well as being appreciated by managers. In the nursing system at tertiary hospitals affiliated to universities of medical sciences, continuous in-service training was also provided for nurses in different job categories. The given training could accordingly improve their job competency and provide a chance for job promotion and better adaptation, and ultimately better WRQoL, which could, in turn, justify the results and the high mean score of job and career satisfaction. Nemati et al. (2020) in a study on levels of awareness in nurses working in hospitals in the city of Shiraz, southern Iran, had further reported that nurses had a high level of awareness of COVID-19. These individuals had generally received this training through hospitals where they were working, the Ministry of Health and Medical Education, and the Internet [22]. This issue could be also raised in explaining the role of work location because working in the hospitals of the city of Shiraz had resulted in a better WRQoL than the ones based in the city of Bushehr as maintained in the study findings. Tertiary hospitals in big cities such as Shiraz were equipped with good medical facilities, specialized teams, and structured work plans, which could provide better conditions for employees’ WRQoL in the current critical situation. In line with these results, Moradi et al. (2014) had also considered hospitals as one of the factors affecting WRQoL [9].

Moreover, stress at work obtained a lower mean score compared with other subscales. The content of the items related to this subscale was associated with stress and pressure on nurses in terms of high-volume and long working hours. Due to the prevalence of COVID-19 and the current critical times, the working situation of nurses in all hospitals, especially those designated as referral centers for these patients in each province, had been severely affected. Pressure, work-related stress, and long working hours with special conditions such as wearing protective clothing and having worries about being infected with the virus had further led to a lower mean score assigned to stress at work among the WRQoL subscales [18]. In agreement with these results, studies by Lu et al. (2020) and Lai et al. (2020) in China had similarly reported high psychological stress and burden on treatment teams, particularly nurses working at the frontlines fighting against COVID-19 [18, 19].

The study results also showed that the nurses had a low mean score in terms of the home-work interface, which could be explained by the fact that more than three-quarters of the participants in this study were female, mostly married. Due to the current critical situation as well as the pressure and long working hours and sometimes hospital stays and not going home to prevent the virus from spreading to other family members, the nurses’ in-house roles had been affected, so this imbalance could influence their WRQoL. With regard to the dominant culture in the cities (southern Iran) examined in this study, wherein women were playing the leading roles at home and they were in charge of doing house chores and caring for children and the elderly, work pressure was greater on working women, particularly nurses with their working conditions and difficulties [28]. With reference to the culture of the Iranian society, the burden of these roles on Iranian female nurses is more than other Western countries, because family members, with a much broader meaning than husband and children, have duties and obligations. From the perspective of traditional society, these responsibilities have been assigned to women [23]. With regard to several studies, pressures cause conflicts between the roles of these women as a spouse or a mother and the duties of an official nurse [29, 30]. Therefore, in Iran, wherein a large number of nurses are women, the position of family in the Iranian culture and Islam is of great importance and work-life balance, especially for married women, is significant. In order to create this balance, it is suggested that better supportive infrastructure be established by the government and the workplace and that legislation be passed to further protect working women.

Furthermore, this study revealed that PSC was an important predictor of WRQoL in nurses. In this regard, Cowin et al. (2008), in their longitudinal study, had found that PSC was a strong predictor of job satisfaction in nurses so that the importance of the role of PSC on the amount of salary paid as an external factor of job satisfaction was more significant with longer stability [11]. Based on other surveys, internal factors such as job satisfaction and PSC could also have an active role in manifestations of symptoms of burnout among nurses, and PSC was a buffer to deal with challenging working conditions [15].

This study had limitations such as its cross-sectional nature, which constrained the interpretation of the causal relationship between the variables in this study. To understand the causal relationship between these variables, it is necessary to design and conduct longitudinal studies. The presence of a group of non-caring nurses in this study to compare them with the group concerned can additionally elucidate the role of caring for patients with COVID-19 in PSC and WRQoL in nurses, which is suggested to be addressed in further investigations.

## Conclusions

In general, PSC is an important determinant of WRQoL in nurses. Given that the global shortages of nurses in health care systems are one of the serious health threats, retaining these individuals in good working conditions and preventing them from quitting jobs is of great importance [10, 11]. Paying attention to nurses’ WRQoL and identifying factors affecting it can thus help these people to stay in their profession. PSC, which is considered as individuals’ perceptions of their job identity, can be correspondingly effective in the type of their professional behavior. Therefore, it is recommended to establish continuous communication between nursing organizations and media such as radio and television and the press as well as the cyberspace such as popular social networks to portray the key role of nurses in health care systems. In this way, professional identity and a better understanding of the nursing profession can be shaped. Providing in-service psychological and professional counseling services, holding training courses to help nurses adapt to their jobs and to gain sufficient skills for a better role, creating a nurse-friendly organizational policy in health care centers, as well as meeting personal and family needs of nurses with a special view towards female nurses responsible for their official job and housework at the same time, can improve WRQoL and provide nurses with better durability to provide high-quality care. Accordingly, public health safety can be enhanced by dealing with crises such as the COVID-19 pandemic.

## Data Availability

The datasets used and/or analyzed during the current study are available from the corresponding author on reasonable request.

## Abbreviations

PSC: Professional Self-Concept
WRQoL: Work-Related Quality of Life
NSCQ: Nurse Self-Concept Questionnaire
